# Letter to the editor: European headache federation guidelines on the use of monoclonal antibodies acting on the calcitonin gene-related peptide or its receptor for migraine prevention

**DOI:** 10.1186/s10194-019-0994-z

**Published:** 2019-06-04

**Authors:** Daniel D. Mikol, Josefin Snellman

**Affiliations:** 1 Amgen Inc., Thousand Oaks, California USA; 2 Novartis Pharma A.G, Basel, Switzerland

To the Editor,

We thank you for publishing the guidelines on the use of monoclonal antibodies (mAbs) targeting the calcitonin gene-related peptide (CGRP) pathway for migraine prevention [1]. We believe that these guidelines will be of great importance for clinicians in guiding treatment decisions and ultimately benefiting patients with migraine, which is a significant contribution to the field.

While reviewing the guidelines, we observed a few inconsistencies in the data presented for erenumab. On Page 20, Fig. 1 mentions “*Treatment with Erenumab 140 mg results in a small unimportant increase of serious adverse events occurrence compared to placebo*” [1]. However, as reflected in Fig. 1, the risk with placebo was 25 per 1000 and 11 per 1000 with erenumab. Hence, there is a “decrease” in serious adverse event occurrence observed with erenumab versus placebo, which we have highlighted in Fig. 1.

**Fig. 1:**
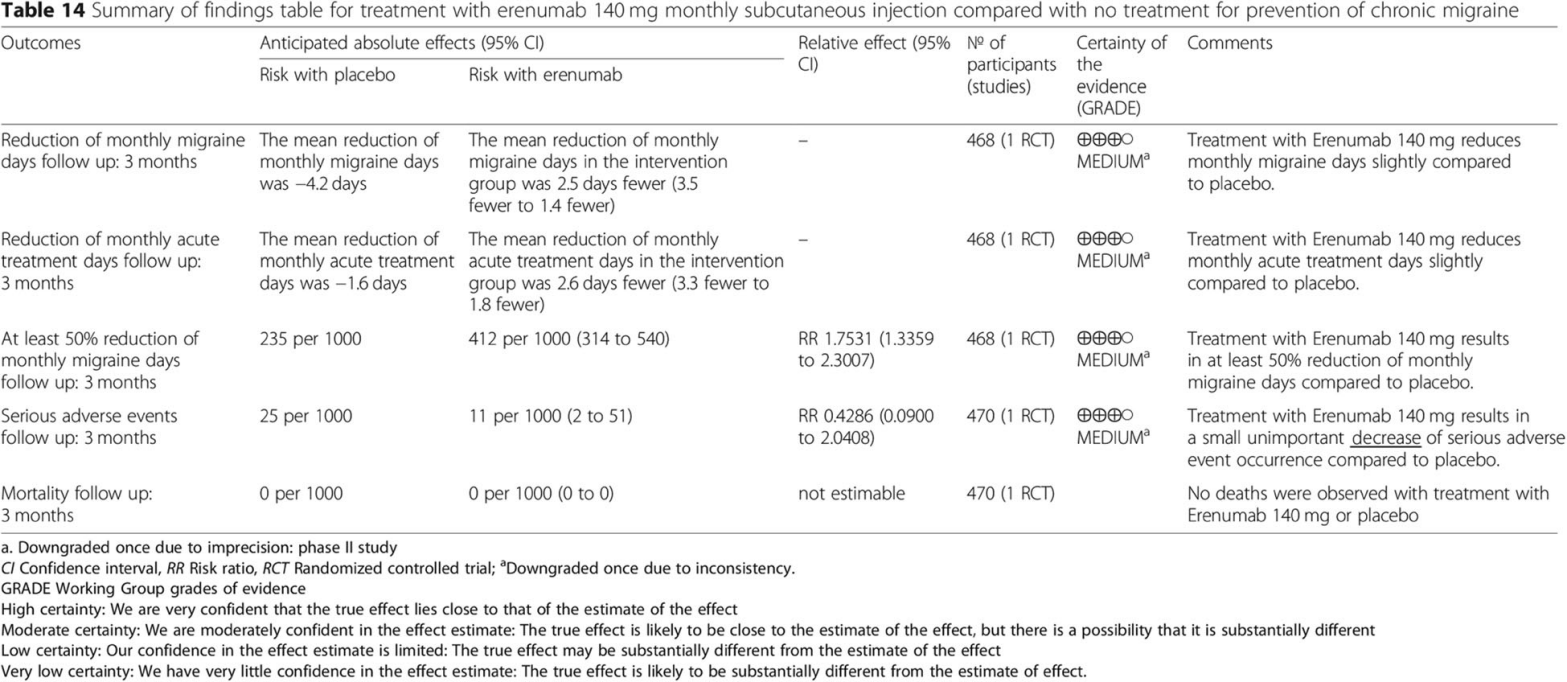
Proposed amends for Table 14

We also observed an inconsistency within Fig. 2 (Page 30) that provides information on binding or neutralising antibodies for all pivotal trials included in this guideline [1]. In this table, the data from the ARISE study [2] have been erroneously shown for the STRIVE study [3]. Similarly, the data from the STRIVE study [3] have been shown for the ARISE study [2]. Also, the percentage of neutralising antibodies in the ARISE study is reported as 0.3%, whereas the correct value is 0.4% (*n* = 1/283). The proposed correction for this swapping of data between the ARISE and STRIVE studies and for correcting the value for the neutralising antibodies is presented in Fig. 2. In addition, Fig. 2 includes frequencies of neutralising antibodies for the 7 mg and 21 mg doses, which were used in a relatively small Phase 2 proof-of-concept study [4]. These doses were ineffective, not studied further, and are not commercially available. Hence, for proper guidance to clinicians, we suggest omitting the data for 7 mg and 21 mg.

**Fig. 2:**
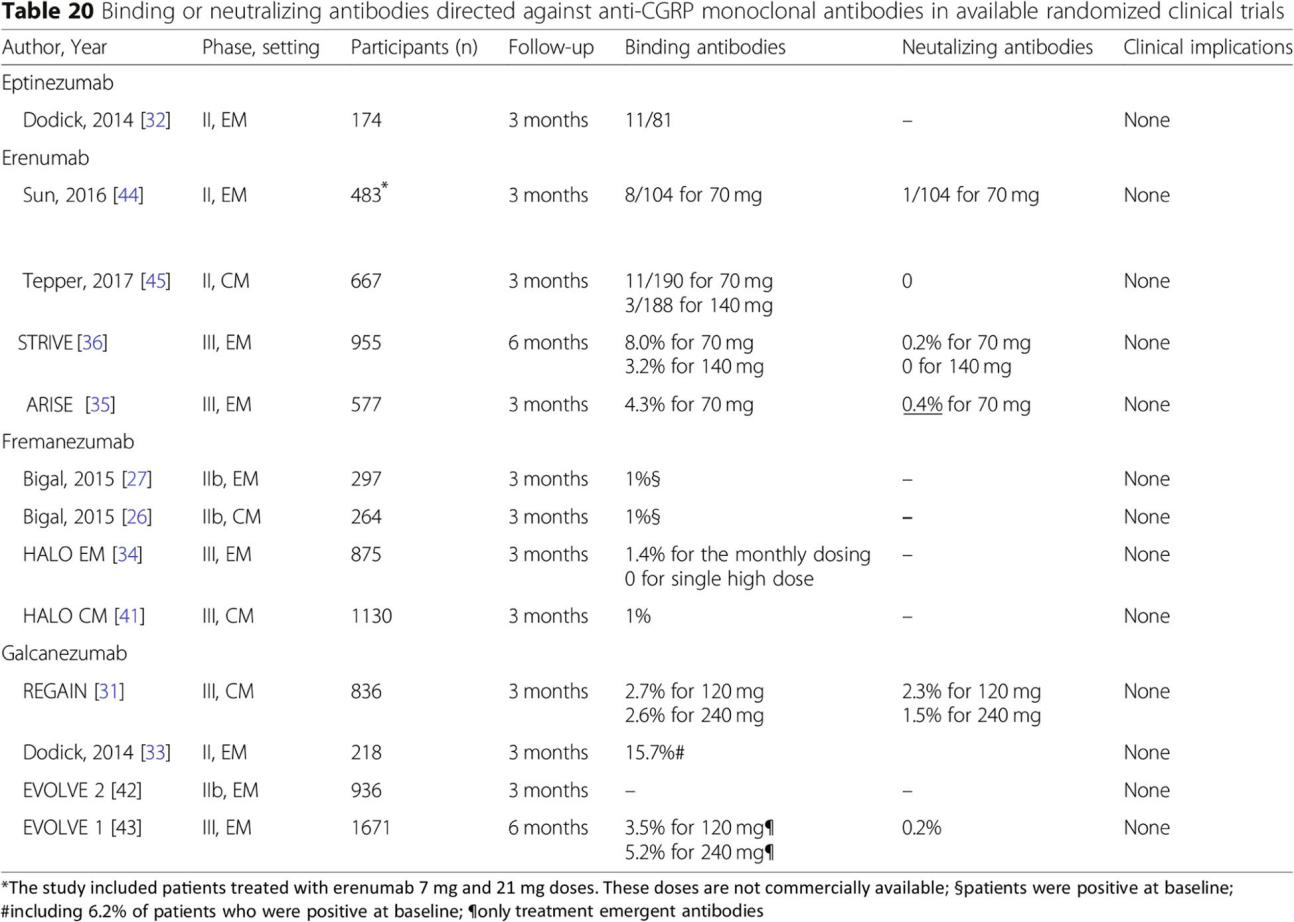
Proposed amends for Table 20

We would like to acknowledge the efforts and contributions of the consensus panel for drafting these guidelines. The data inconsistencies highlighted in this letter could have affected the final results and recommendations made in the guidelines. Moreover, the erroneous data may be cited by authors in upcoming publications, which may potentially affect the recommendations for the mAbs targeting the CGRP pathway. Hence, we request that you consider the proposed amendments to address these inconsistencies for the benefit of the readers.

## Abbreviations

CGRP: Calcitonin gene-related peptide
mAb: Monoclonal antibody
